# Model-Based Hybrid Control of Pure Pursuit and Stanley Methods for Vehicle Path Tracking

**DOI:** 10.3390/s25206491

**Published:** 2025-10-21

**Authors:** Hojin Jung

**Affiliations:** Department of Automotive Engineering, Korea National University of Transportation, Chungju 27469, Republic of Korea; hojin.jung@ut.ac.kr

**Keywords:** autonomous vehicle, hybrid control, path-tracking, Pure Pursuit, Stanley, interactive multiple-model Kalman filter

## Abstract

In this study, a new method was applied to systematically combine the two controllers, which can help overcome the limitations of non-systematic combinations such as rule-based methods. For the model-based process, the bicycle model was used. Then, the model probability was calculated through the interactive multiple model filtering algorithm, which stochastically determines the most appropriate model that fits the current dynamic situation of the vehicle well. Based on this result, a hybrid path tracking controller was developed using the model probability of each method. The superiority of the proposed method was validated using the MORAI Drive simulator, which reflects the real road environment well enough. The results showed that the RMS tracking performance error was reduced by 6.0–8.8% in quarter-circle path and 3.3% in general path compared to single methods.

## 1. Introduction

In recent years, GPS, IMU, lidar, and camera sensors have been used in research to control autonomous vehicles, and various methods of sensor fusion have been developed. Especially with the increasing application of AI technology in the field of autonomous vehicles, the development of control architecture for autonomous driving has evolved from the traditional perception-decision-control pipeline toward end-to-end learning-based control architectures, which has become a new trend. Although the trend is changing rapidly, path-tracking controllers that make the vehicle follow predetermined path coordinates using GPS and IMU sensors as feedback are still commonly used as the primary algorithm for autonomous vehicles [1], which leads to research for developing advanced path planning [2] and smart roundabouts for traffic optimization [3].

In general, path tracking methods can be divided into two main approaches. First, geometric methods use the geometric relationship between the path and the vehicle to calculate a target wheel steer angle that can be used to follow the path. Among several methods, Pure Pursuit [4] and Stanley [5] methods are the most well-known. Secondly, in the model-based method, the target wheel steer angle is calculated using a mathematical model that expresses the motion of the vehicle. This has the advantage that various advanced controllers can be developed by applying classical control theories. Most of the latest model-based approaches consist of the MPC method [6,7,8], but compared to the geometric method, it has the limitation that it is difficult to be used as a real-time controller due to dependence on model parameters, limited robustness, and high computational complexity required for optimal control performance [9].

Although geometric methods are more suitable for real-time controller design than model-based methods, each method also has certain advantages and disadvantages. The Pure Pursuit method can show good proximity tracking performance if the look-ahead distance, which functions as a proportional gain, is determined appropriately. If the look-ahead distance is too large, the tracking response becomes smoother, but corner cutting may occur, thus greatly reducing tracking accuracy and safety in some cases. If the look-ahead distance is too small, the vehicle tends to track the path more accurately, but steering oscillations will occur due to the rapid change of control input. The Stanley method can show a fast approach to the target path by using the crosstrack error. However, this method also leads to large path tracking errors if the target path is not smooth. To overcome the disadvantages of each geometric method, there have been many previous studies. For example, the use of variable parameters was a basic option [10]. An improved curve fitting method was used to reduce the tracking error [11]. An adaptive controller using a fuzzy supervisory system has also been addressed [12,13]. A predictive concept that merges the results of multiple processes has also been proposed [14]. However, these approaches only improve the performance of the individual geometric methods themselves and therefore cannot combine the advantages of the individual methods. There are also several studies on the hybrid controller for autonomous vehicles. Cibooglu et al. [15] proposed a hybrid algorithm of Pure Pursuit and Stanley method. However, their approach is heuristic and relies only on the rule-based algorithm. Cao et al. [16] also proposed a fusion algorithm of the two methods, but its main algorithm was simply designed with a switching function between the two methods based on thresholds and only showed validation results for indoor environments. Zhang et al. [17] proposed an adaptive path tracking algorithm of Pure Pursuit and PID methods. Although this study used an ant colony optimization technique in order to optimize the weighing factors of two methods, it still remains a challenge to weight the feed-forward and feed-back terms to accommodate various tracking scenarios.

To summarize, previous research on hybrid controllers has not systematically addressed the problem. Therefore, in this paper, we propose an algorithm that systematically combines two methods. As a solution, the bicycle model was chosen as the processing model in this paper to represent the dynamic characteristics of the vehicle and overcome the limitations of the purely geometric method. The main contributions of this study can be summarized as follows:

- A probabilistic hybrid control framework that integrates the Pure Pursuit and Stanley controllers based on real-time model probabilities derived from an IMM filter.
- Adopting a bicycle model that helps to represent the actual dynamic behavior of the vehicle for proper geometric method selection.

The remainder of this paper is structured as follows: Section 2 reviews related work in geometric methods and bicycle models. Section 3 describes the proposed IMM-based control framework in detail. Section 4 presents the simulation tool, the conditions for setting up sensors and controllers, and the scenarios for validation. Section 5 analyzes the results of the simulations in detail. Finally, Section 6 concludes the study and discusses future research directions.

## 2. Related Work

### 2.1. Pure Pursuit Method

**Figure 1:**
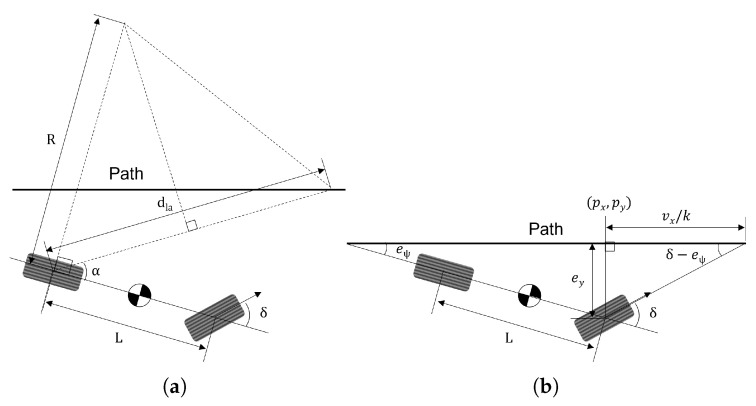
Schematic representation of the geometric method for path tracking. (**a**) Pure Pursuit. (**b**) Stanley.

As mentioned earlier, Pure Pursuit is a well-known algorithm for path-tracking and has already been presented in many studies. Figure 1a shows the geometrical representation of the Pure Pursuit method. The core idea of the Pure Pursuit method is that it always searches for a target point on the trajectory and finds the wheel steer angle that leads the vehicle through the target point. It is assumed that the motions of the vehicle are governed by vehicle kinematics, i.e., no tire slip occurs.

Using the law of sines, the following equation can be derived:

(1) $$\sin\left(\alpha \right)=\frac{d_{la}}{2R}$$

where α is the look-ahead heading, defined by the angle between the vehicle heading and the line connecting the rear axle of the vehicle to the target point. $d_{la}$ is the look-ahead distance that is the distance between the vehicle’s rear axle and the target point, and *R* is the turning radius determined by the vehicle kinematics. Using the Ackermann angle [18] and (1), the following equation is obtained:

(2) $$\tan\left(\delta \right)=\frac{L}{R}=L\frac{2\sin(\alpha )}{d_{la}}$$

where *L* is the length of the wheelbase.

Finally, the desired wheel steering angle of the Pure Pursuit method, $\delta_{p}$ is determined as follows:

(3) $$\delta_{p}=\tan^{-1}\left(\frac{2L\sin(\alpha )}{d_{la}}\right)$$

It is known from (3) that the look-ahead distance is the only parameter. If a large look-ahead distance is selected, the steer response becomes smooth, but tracking performance deteriorates, and the cutting corner problem may occur. If a small look-ahead distance is selected, the steering response will oscillate, which can lead to instability in path tracking. Therefore, a velocity-dependent look-ahead distance is usually used for optimization [19]. The following equation was used in this study:

(4) $$d_{la}=max\left(5,k_{d}v_{x}\right)$$

where $k_{d}$ is the coefficient used to adjust the look-ahead distance. $v_{x}$ is the longitudinal speed of the vehicle. Also, it is noteworthy that a minimum look-ahead distance of 5 m is taken in order to avoid the sensitivity of $k_{d}$ in low speeds. Here, the unit of $k_{d}$ is seconds.

### 2.2. Stanley Method

Stanley is another popular algorithm for path-tracking that has been addressed in many studies. Figure 1b shows the geometrical representation of the Stanley method. In this method, both the crosstrack error and heading angle error are used to calculate the wheel steer angle without considering the look-ahead distance. Using the trigonometric relation shown in Figure 1b, the following equation can be derived:

(5) $$\tan\left(\delta -e_{\psi }\right)=\frac{ke_{y}}{v_{x}}$$

where $e_{\psi }$ is the heading angle error, $e_{y}$ is the closest distance from the center of the front wheel to the target path point $\left(p_{x},p_{y}\right)$, i.e., the crosstrack error. And *k* is the coefficient used to adjust the target path speed. Here, the unit of *k* is the reciprocal of a second.

By rearranging (5), the desired wheel steering angle of the Stanley method, $\delta_{s}$ is obtained as follows:

(6) $$\delta_{s}=e_{\psi }+\tan^{-1}\left(\frac{ke_{y}}{v_{x}}\right)$$

In (6), using the crosstrack error directly helps the vehicle to follow the target path quickly. However, if the path contains discontinuous points, it shows an unstable response because it has large tracking errors. In general, it is known that the Stanley method is suitable for high-speed driving.

### 2.3. Bicycle Model

**Figure 2:**
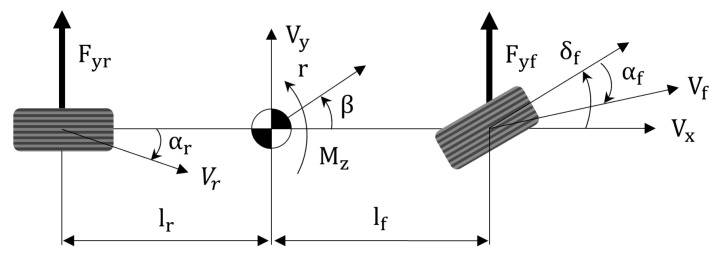
Bicycle model.

The bicycle model is a popular model in the field of vehicle dynamics. Although it has a rather simple structure, it can describe the dynamic behavior of vehicles. Due to this strength, it has been applied in many studies for the design of vehicle dynamics controllers [20,21,22]. Based on the relationship between force and state shown in Figure 2, the following state-space form is obtained:

(7) $$\left[\begin{array}{c} \dot{\beta }\\ \dot{r} \end{array}\right]=\left[\begin{array}{cc} -\frac{2(C_{f}+C_{r})}{mv_{x}} & \frac{-2(C_{f}l_{f}-C_{r}l_{r})}{mv_{x}^{2}}-1\\ \frac{-2(C_{f}l_{f}-C_{r}l_{r})}{I_{z}} & \frac{-2(C_{f}l_{f}^{2}+C_{r}l_{r}^{2})}{I_{z}v_{x}} \end{array}\right]\left[\begin{array}{c} \beta \\ r \end{array}\right]+\left[\begin{array}{c} \frac{2C_{f}}{mv_{x}}\\ \frac{2C_{f}l_{f}}{I_{z}} \end{array}\right]\delta_{f}$$

where $\beta ,r,C_{f},C_{r},l_{f},l_{r},I_{z}$, and *m* denote the sideslip angle of the vehicle, yaw rate, front cornering stiffness, rear cornering stiffness, the distance between the center of mass of the vehicle and the front axle, the distance from the center of mass of the vehicle and the rear axle, the wheel yaw moment of inertia and the vehicle mass. Here, this bicycle model is used as a representative model for Pure Pursuit and Stanley, by replacing the input value of $\delta_{f}$, the front wheel steering angle, with the values of $\delta_{p}$ and $\delta_{s}$, respectively.

## 3. Method

In the previous section, the derivation of the desired wheel steer angle of the Pure Pursuit and Stanley methods for path tracking was examined. Then, the applicability and feasibility of the bicycle model, which was selected in this study for model-based processing, were explained. Now, the method for systematic calculation for model fusion is presented in this section.

### 3.1. IMM Filter Design

**Figure 3:**
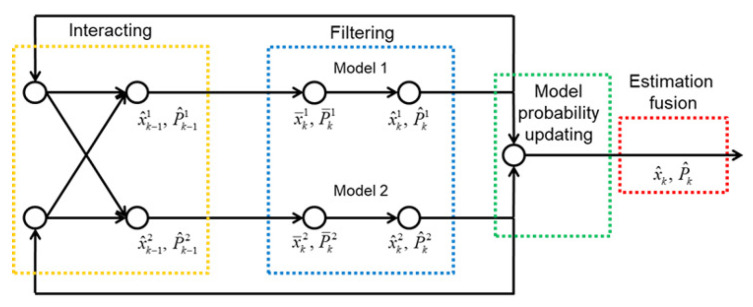
Schematic of IMM filter.

The IMM filter can be a solution for model fusion when the current system is governed by several dynamic models. By applying the model probability, which is determined based on a stochastic filtering process, as a weighting factor, the IMM filter can logically integrate the results of each model. Its effectiveness has already been proven in many areas of automotive engineering [23,24,25,26]. Figure 3 shows the schematic of the IMM filtering process.

For the state vector used in IMM filtering, the sideslip angle and yaw rate, which are state variables of the bicycle model, were used. It is also worth mentioning that this filtering was implemented assuming full-state feedback from GPS and IMU sensors.

#### 3.1.1. Interacting

Using the values of the associated state vector, probability, and covariance of each model from the previous step, the IMM filter calculates the initial mixed values for the calculation of the current step. First, the mixed probability $\bar{p}$ is determined as follows:

(8) $${\bar{p}}_{k}^{i}=\sum_{j=1}^{2}H_{ji}{\hat{p}}_{k-1}^{j},i=1,2$$

where $H_{ji}$ is the probability transition matrix, which indicates the probability that the vehicle dynamics model will transition from model *j* to *i*. The index number 1 indicates the bicycle model of Pure Pursuit, and 2 denotes that of Stanley. *k* and k−1 denote the current and previous step, respectively. ${\hat{p}}_{k-1}$ is the probability of each model of previous step. Considering the empirical findings and the lessons from previous studies that Pure Pursuit is reliable at low speed and Stanley is vice versa, the probability transition matrix can be designed as follows:

(9) $$\left\{\begin{array}{c} H_{11}=0.9-min\left(v_{x}/60,1\right)/10\\ H_{12}=0.1+min\left(v_{x}/60,1\right)/10\\ H_{21}=0.95-min\left(v_{x}/60,1\right)/10\\ H_{22}=0.05+min\left(v_{x}/60,1\right)/10 \end{array}\right.$$

Here, it is worth noting that the value of the probability transition matrix is biased in favor of the Pure Pursuit method, as the results show that the overall tracking performance of the Stanley method was worse than that of Pure Pursuit. Therefore, in order to use the characteristics of the Stanley method only on a high curvature path, the model probability was fixed to 1 for Pure Pursuit and 0 for Stanley when the local path radius is greater than 50 m. A more detailed analysis is discussed in Section 5.

With the value of the mixed probability, the initial mixed state vector $\bar{x}$ and the covariance matrix $\bar{P}$ of each model are as follows:

(10) $${\bar{x}}_{k-1}^{i}=\sum_{j=1}^{2}H_{ij}{\hat{p}}_{k-1}^{j}{\hat{x}}_{k-1}^{j}/{\bar{p}}_{k}^{i}$$

(11) $${\bar{P}}_{k-1}^{i}=\sum_{j=1}^{2}H_{ij}{\hat{p}}_{k-1}^{j}\left[{\hat{P}}_{k-1}^{j}+\left({\hat{x}}_{k-1}^{j}-{\bar{x}}_{k-1}^{i}\right){\left({\hat{x}}_{k-1}^{j}-{\bar{x}}_{k-1}^{i}\right)}^{T}\right]/{\bar{p}}_{k}^{i}$$

#### 3.1.2. Filtering

With the state vector and covariance matrix of each model resulting from the interaction process, Bayesian filtering of each model is performed at this stage. In this study, the discrete-time Kalman filtering algorithm [27], which is the most common and useful for linear systems, was used.

- Process update

Based on the process models, *a priori* estimates of the state and covariance of the current step can be obtained. The process update procedures are as follows:

(12) $${\hat{x}}_{k-}^{i}=F_{k-1}^{i}{\bar{x}}_{k-1}^{i}+G_{k-1}$$

(13) $${\hat{P}}_{k-}^{i}=F_{k-1}^{i}{\bar{P}}_{k-1}^{i}{\left(F_{k-1}^{i}\right)}^{T}+Q$$

Here, (12) is the discretized form of each continuous process model. ***Q*** is the process noise covariance matrix.

- Measurement update

By applying the measurement $y_{k}$ in a feedback correction, *a posteriori* estimates of the state and covariance of the current step can be obtained. The procedures for updating the measurement are as follows:

(14) $$K_{k}^{i}={\hat{P}}_{k-}^{i}H^{T}{\left(H{\hat{P}}_{k-}^{i}H^{T}+R\right)}^{-1}$$

(15) $${\hat{x}}_{k+}^{i}={\hat{x}}_{k-}^{i}+K_{k}^{i}\left(y_{k}-H{\hat{x}}_{k-}^{i}\right)$$

(16) $${\hat{P}}_{k+}^{i}=\left(I-K_{k}^{i}H\right){\hat{P}}_{k-}^{i}$$

where ***H*** is the measurement matrix of *y* and ***R*** is the sensor noise covariance matrix.

Considering the noise level of the sensors and model uncertainties, the values of the noise covariance matrices were selected as follows:

(17) Q=diag[0.1,0.01]

(18) R=diag[0.001,0.001]

Here, it is noteworthy that the sideslip angle sensor accuracy is lower than that of the yaw rate sensor due to the indirect calculation from the GPS sensor. Also, it was assumed that model uncertainty is minor compared with sensor uncertainty.

#### 3.1.3. Model Probability Updating

Based on the a posteriori estimates of each model resulting from the filtering stage, a probability update is then performed. Assuming that the process noise and the measurement noise of each model are Gaussian, the likelihood function is defined by the following equation:

(19) $$\Gamma_{k}^{i}=\frac{\exp\left(-\frac{1}{2}{\left(v_{k}^{i}\right)}^{T}{\left(S_{k}^{i}\right)}^{-1}v_{k}^{i}\right)}{\sqrt{\left|2\pi S_{k}^{i}\right|}}$$

where $v_{k}^{i}$ and $S_{k}^{i}$ are the innovation terms of measurement and covariance, respectively, defined as follows:

(20) $$v_{k}^{i}=y_{k}-H{\hat{x}}_{k-}^{i}$$

(21) $$S_{k}^{i}=H{\hat{P}}_{k-}^{i}H^{T}+R$$

Then, the probability of model *i* at the current step is given by the following:

(22) $${\hat{p}}_{k}^{i}=\frac{{\bar{p}}_{k}^{i}\Gamma_{k}^{i}}{\sum_{i=1}^{2}{\bar{p}}_{k}^{i}\Gamma_{k}^{i}}$$

#### 3.1.4. Estimation Fusion

By applying the associated probability of each model as a weighting factor, the final mixed state vector and the covariance matrix can be determined as follows:

(23) $${\hat{x}}_{k}=\sum_{i=1}^{2}{\hat{p}}_{k}^{i}{\hat{x}}_{k}^{i}$$

(24) $${\hat{P}}_{k}=\sum_{i=1}^{2}{\hat{p}}_{k}^{i}\left[{\hat{P}}_{k}^{i}+\left({\hat{x}}_{k}^{i}-{\hat{x}}_{k}\right){\left({\hat{x}}_{k}^{i}-{\hat{x}}_{k}\right)}^{T}\right]$$

The final result of (23) and (24) is the initial values for the interaction stage in the next step. The main purpose, or reason for using the IMM filter in this study, is not the state estimation, but the application of the model probability. Therefore, the modified desired wheel steer angle for path tracking in the current step can be derived from the following equation:

(25) $$\delta_{m}={\hat{p}}_{k}^{1}\delta_{p}+{\hat{p}}_{k}^{2}\delta_{s}$$

## 4. Simulation Setup

**Figure 4:**
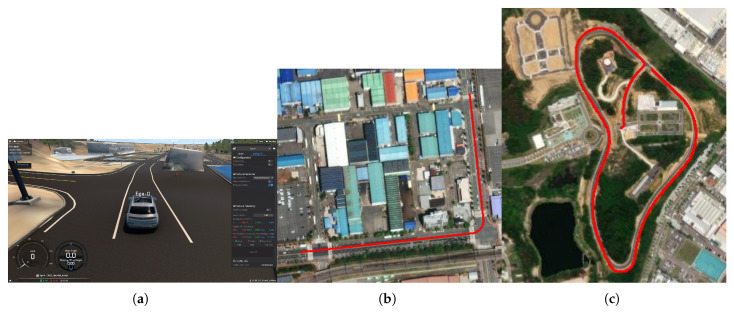
Image of the simulator and selected path for controller validation. (**a**) Basic environment of the simulator. (**b**,**c**) Visualization of the driving on a satellite image.

**Figure 5:**
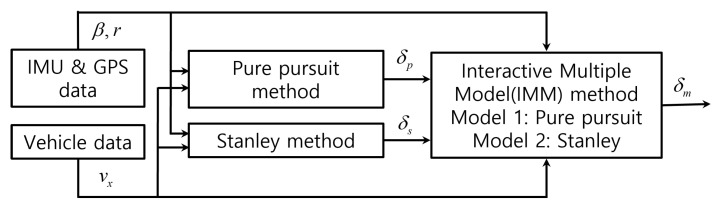
Block diagram of hybrid control of Pure Pursuit and Stanley methods.

Simulations were carried out to verify the effectiveness of the proposed algorithm. Among several simulators available for autonomous vehicles, such as CARLA [28], Prescan [29], and MORAI Drive [30], was used in this study. This simulator contains digital twin data of the real environment, which implies that it can virtually simulate the vehicles with a map of the real world, thus increasing the feasibility and reliability of the simulation results. The simulations were conducted in two steps. The first is the tracking on the quarter-circle path. In this simulation, the control response for each path tracking method and the proposed method was investigated and compared when the vehicle tracks a quarter-circle path of a radius of 20 m while driving in a straight line at a constant speed. Secondly, tracking on the normal road was investigated while the vehicle was travelling at a variable speed. The last simulation was performed to analyze the feasibility of the proposed algorithm under general conditions. For the first simulation, a digital twin map that exists in Korea at 126.7649 of longitude and 35.1246 latitude was selected. The total length of this path is 506 m. For the second simulation, a digital twin map of about 600 m by 300 m in size was selected, located in Korea at 127.4430 longitude and 36.7274 latitude. The total length of this path is 1595 m. Starting from the departure point, it includes curved sections with road turning radii of approximately 15 m, 50 m, 80 m, and 30 m. Immediately before the arrival point, there is a straight section approximately 130 m in length. During the simulations, it was assumed that obstacles did not exist and trajectories were known with enough waypoint data. Figure 4a shows the main screen of the simulator. Figure 4b,c show the selected map and path plotted on the satellite image for each simulation. These images are aligned with east to the right. Table 1 contains the values of the parameters of the bicycle model. The vehicle type used in the simulation was a sedan. Table 2 contains the values of parameters used in the path tracking methods. In order to reduce the influence of other control variables on verifying the proposed algorithm, the sampling conditions were set fast enough. The sampling time of the simulation was set to 40 Hz, and the sampling time of the sensors was set to 10 Hz for GPS and 40 Hz for IMU, respectively. Figure 5 shows the block diagram of the proposed algorithm. Here, the sideslip angle β of the vehicle, which is used in the IMM method for sensor feedback, was obtained through post-processing from the raw data of the GPS and IMU sensors. In the simulation, the ROS communication method, which is commonly used for the design of an autonomous controller, was applied. The suggested algorithm was simulated using an Intel i5-12500 processor (3 GHz). Simulations under identical conditions were performed three times, and the mean value was used for data analysis.

As with the speed planning in the second simulation, a simple rule-based algorithm was used. The target speed, $v_{xd}$, of the vehicle was determined based on the radius of the local path, given by the following equation:

(26) $$v_{xd}=\left\{\begin{array}{c} 20\;\mathrm{km}/h,\;\mathrm{if}\;\mathrm{path}\;\mathrm{radius}<50\;m\\ 40\;\mathrm{km}/h,\;\mathrm{if}\;50\;m<\mathrm{path}\;\mathrm{radius}<200\;m\\ 60\;\mathrm{km}/h,\;else \end{array}\right.$$

## 5. Results and Discussions

### 5.1. Path Tracking on a Road with a Quarter-Circle Path

**Figure 6:**
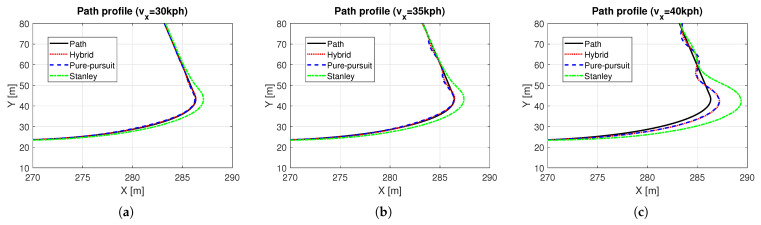
Vehicle path trajectories near the quarter-circle area: (**a**) $v_{x}$ = 30kph. (**b**) $v_{x}$ = 35 kph. (**c**) $v_{x}$ = 40 kph.

**Figure 7:**
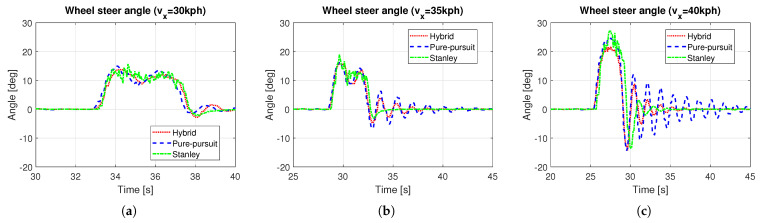
Wheel steer angle in dominant time ranges: (**a**) $v_{x}$ = 30 kph. (**b**) $v_{x}$ = 35 kph. (**c**) $v_{x}$ = 40 kph.

**Figure 8:**
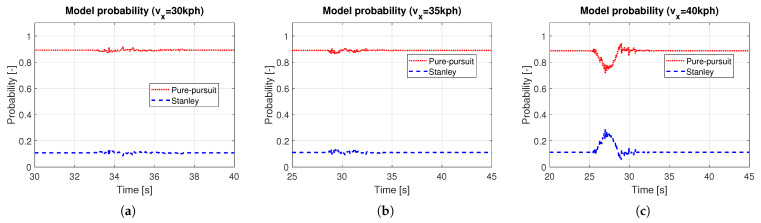
Model probabilities of IMM filter: (**a**) $v_{x}$ = 30kph. (**b**) $v_{x}$ = 35 kph. (**c**) $v_{x}$ = 40 kph.

Based on the methods presented in Section 3, a hybrid controller was designed, and simulations were performed. In this simulation, a quarter-circle turning while driving on a straight line with constant speed was performed. For the object analysis as a function of vehicle speed, several simulations with different constant target speeds were carried out. Figure 6 shows the path tracking results depending on vehicle speed, especially in the ranges near the quarter-circle path. The black solid line is the reference path, the red dotted line is the path of the hybrid controller, the blue dashed line is the path of the Pure Pursuit method, and the green dash-dot line is the path of the Stanley method, respectively. It was evident that the performance of the path tracking deteriorates as the vehicle speed increases, which is due to the effects of the lateral dynamics of the vehicle. The Stanley method showed an out-cornering tendency regardless of the vehicle entry speed of cornering, while this was not the case with Pure Pursuit. Overall, the tracking performance of Pure Pursuit was better than that of the Stanley method. Due to the biased setting of the probability transition matrix, which gives more weight to the Pure Pursuit method (see (9)), the tracking performance of the hybrid controller was more analogous to the Pure Pursuit method. Figure 7 shows the response to the control input. Here, time ranges were adjusted for careful verification of the transient response. At a vehicle speed of 30 kph, the two methods are not very different. However, when the vehicle speed is above 30 kph, the Pure Pursuit method shows an unstable response immediately after the quarter-circle turn. This phenomenon becomes severe as the vehicle speed increases (see t=33–40 s of Figure 7b and t=30–42 s of Figure 7c). The Stanley method showed a stable controller response even after the quarter-circle turn. Finally, the hybrid controller showed an oscillatory response at a level between Pure Pursuit and the Stanley method. However, its control input is not always a value between Pure Pursuit and Stanley, especially when the tire lateral force or sideslip angle increases. In Figure 7c, the initial wheel steer angle of the hybrid controller was lower than that of the Pure Pursuit and the Stanley method (see t=25–29 s of Figure 7c). This is because the initial response time of the Pure Pursuit and Stanley methods was slightly different (see t=25 s of Figure 7c and investigate the start time of cornering between Pure Pursuit and Stanley). Thus, the hybrid controller had a completely different response, which helped to improve the accuracy of path tracking. Figure 8 shows the model probabilities obtained from the IMM filter. The red dotted line is the probability of the Pure Pursuit method, and the blue dashed line is that of the Stanley method. There are two notable meanings of the probabilities: First, although the weights of the two methods are far from being half and half, the hybrid controller may react completely differently from the two methods. Second, the values of the model probabilities changed in a range where the vehicle is cornering, of which the vehicle is affected by lateral dynamics (see t=32–33 s of Figure 8a, t=30–31 s of Figure 8b, and t=28–29 s of Figure 8c). Moreover, the changed values of the model probabilities became larger when the lateral motion of the vehicle became stronger, which proves that the bicycle model operates effectively within the IMM filter framework.

Table 3 shows the result of the tracking error as a function of vehicle speed. Here, the tracking error of the Stanley method was the largest. The Pure Pursuit method had a fairly reasonable tracking error, although its response was unstable at the end of the cornering. The proposed algorithm using the hybrid concept had slightly better tracking performance, and its improvement was more effective when the vehicle had a high speed.

### 5.2. Path Tracking on Normal Road

**Figure 9:**
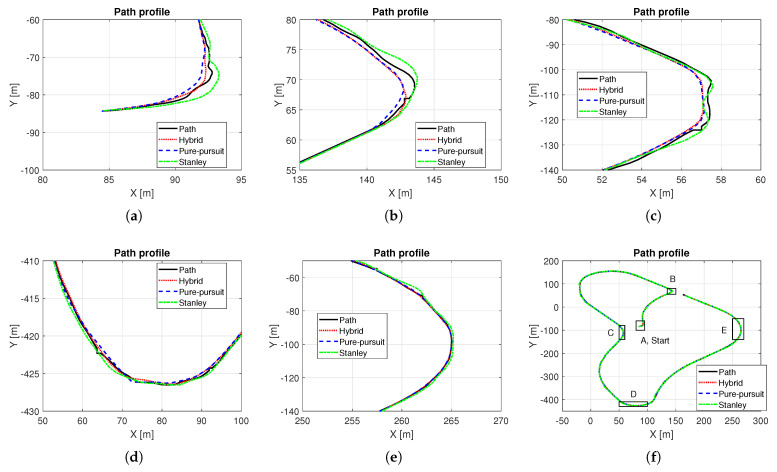
Path trajectories and their enlarged view: (**a**) Enlarged plot of range A. (**b**) Enlarged plot of range B. (**c**) Enlarged plot of range C. (**d**) Enlarged plot of range D. (**e**) Enlarged plot of range E. (**f**) Total path.

**Figure 10:**
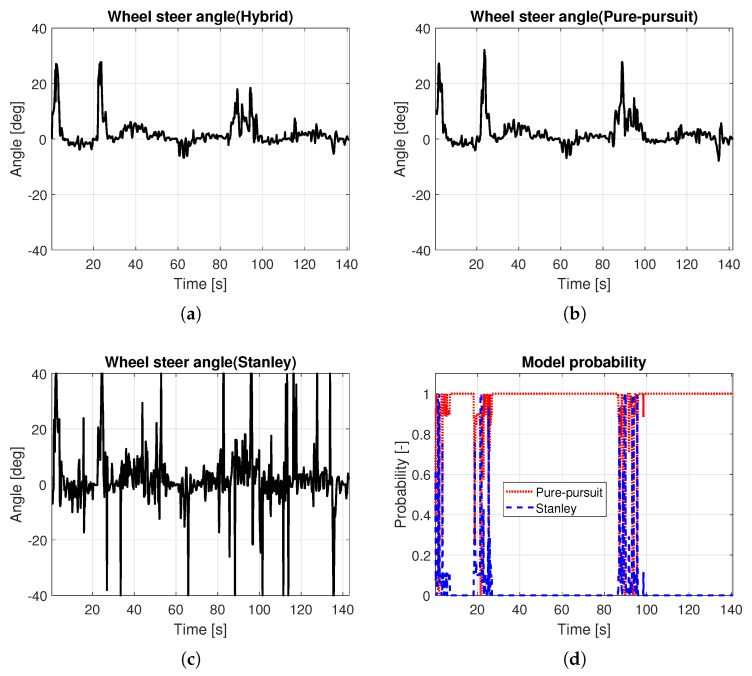
Controller inputs and calculated results of the IMM filter. (**a**) Wheel steer angle (Hybrid). (**b**) Wheel steer angle (Pure Pursuit). (**c**) Wheel steer angle (Stanley). (**d**) Model probabilities.

In this simulation, the proposed algorithm was verified on a normal road. Figure 9f shows the total path. In range A, the first cornering range, the in-corner response appeared in Pure Pursuit, and the out-corner response appeared in Stanley. Using the characteristics of the two methods by representing the vehicle dynamics behavior obtained from the IMM filter, the hybrid controller had a better response than the two methods, by tracking the desired path more accurately (see Figure 9a). A similar phenomenon also occurred in range B (see Figure 9b). In ranges C and E, where the calculated path radius is larger than 50 m, the hybrid controller showed almost the same response as Pure Pursuit (see Figure 9c,d). In range D, where the calculated path radius is smaller than 50 m, the hybrid controller is also effective, as it has better path tracking performance compared to Pure Pursuit and Stanley (see Figure 9e). To summarize, when a hybrid controller is used, it has a tendency to maintain its response between that of the Pure Pursuit and Stanley methods. Figure 10a–c show the wheel steer angle of each method. In this map, Pure Pursuit had a fairly stable response, but Stanley did not, as the waypoint data is irregular. As the current hybrid controller was designed to follow the Pure Pursuit method under the condition of a low-curvature path, the input of the hybrid controller in this simulation is more analogous to the Pure Pursuit method. However, when combined values are used, the hybrid controller has a slightly lower wheel steer angle. In other words, the hybrid controller showed a more stable response than the Pure Pursuit method, while achieving better tracking performance. Figure 10d shows the model probabilities. Under the condition of a small radius, where the vehicle can be influenced by dynamics, rather than kinematics, the model probability operation was processed, and thus gave a quantitative indication of which methods are more suitable in the current situation. t=0–9 s, t=17–28 s, and t=85–99 s of Figure 10d affected the hybrid controller of ranges A, B, and D, respectively. Table 4 is the result of the tracking error of the whole path. Here, the tracking error of the Stanley method was the worst. Pure Pursuit had a better tracking performance than Stanley. In conclusion, the hybrid controller shows the best tracking performance as it has a lower tracking error than Pure Pursuit in the ranges where the combined wheel steer angle is used.

## 6. Conclusions

This study investigated a hybrid controller combining the Pure Pursuit and Stanley methods. The main contributions are the use of a bicycle model to represent the vehicle dynamic characteristics and the systematic approach for a hybrid controller that can improve path tracking performance using the model adopted. In order to apply the systematic method, an IMM filter, which is one of the Kalman filter-based fusion algorithms, was designed. To demonstrate both the feasibility and applicability of the proposed method, validation was performed under two different simulation conditions. The first simulation verified whether the hybrid controller exhibits superior performance compared to conventional controllers when cornering in a quarter-circle while driving in a straight line. Here, the RMS tracking error was reduced by 6.0–8.8%. Furthermore, the proposed method was additionally verified under more general road conditions. Here, the RMS tracking error was reduced by 3.3% due to the fact that there exist ranges where the simultaneous combination of both methods could improve the tracking performance. However, the improvement effect of the proposed method was quite limited. Further productive studies related to this research are expected. Initially, models that better reflect the vehicle dynamics characteristics beyond the bicycle model could be used. A combination with path tracking methods other than Pure Pursuit and Stanley is also possible. In addition, the establishment of a vehicle test environment to obtain experimental results on real vehicles is expected to strengthen the reliability of the results of this study.

## Figures and Tables

**Table 1:** Parameter values of the bicycle model.

Parameter	Value	Parameter	Value
*m*	2000 kg	$C_{f}$	60,000 N/rad
$I_{z}$	4000 kg·m^2^	*C_r_*	60,000 N/rad
*L*	3 m	$l_{f}$	1.5 m

**Table 2:** Parameter values used in the Pure Pursuit and Stanley methods.

Parameter (Pure Pursuit)	Value	Parameter (Stanley)	Value
$k_{d}$	0.5 s	*k*	1/s

**Table 3:** RMS values of distance error for quarter-circle turn.

Tracking Error	Pure Pursuit	Stanley	Hybrid
$v_{x}$ = 30 kph	0.125	0.302	0.114
$v_{x}$ = 35 kph	0.138	0.387	0.126
$v_{x}$ = 40 kph	0.301	0.769	0.283

The unit of data is the meter.

**Table 4:** RMS values of distance error for normal road.

	Pure Pursuit	Stanley	Hybrid
Tracking error	0.481	0.643	0.465

The unit of data is the meter.

## Data Availability

The original contributions presented in this study are included in the article. Further inquiries can be directed to the corresponding author.

## Abbreviations

The following abbreviations are used in this manuscript:

GPS	Global Positioning System
IMM	Interactive Multiple Model
IMU	Inertial Measurement Unit
MPC	Model Predictive Control
RMS	Root Mean Square
ROS	Robot Operating System
